# FormulationAI: a novel web-based platform for drug formulation design driven by artificial intelligence

**DOI:** 10.1093/bib/bbad419

**Published:** 2023-11-22

**Authors:** Jie Dong, Zheng Wu, Huanle Xu, Defang Ouyang

**Affiliations:** Xiangya School of Pharmaceutical Sciences, Central South University, Changsha, China; Institute of Chinese Medical Sciences (ICMS), State Key Laboratory of Quality Research in Chinese Medicine, University of Macau, Macau, China; Institute of Chinese Medical Sciences (ICMS), State Key Laboratory of Quality Research in Chinese Medicine, University of Macau, Macau, China; Faculty of Science and Technology, University of Macau, Macau, China; Institute of Chinese Medical Sciences (ICMS), State Key Laboratory of Quality Research in Chinese Medicine, University of Macau, Macau, China

**Keywords:** FormulationAI, artificial intelligence, computational pharmaceutics, drug formulation, drug design, webserver

## Abstract

Today, pharmaceutical industry faces great pressure to employ more efficient and systematic ways in drug discovery and development process. However, conventional formulation studies still strongly rely on personal experiences by trial-and-error experiments, resulting in a labor-consuming, tedious and costly pipeline. Thus, it is highly required to develop intelligent and efficient methods for formulation development to keep pace with the progress of the pharmaceutical industry. Here, we developed a comprehensive web-based platform (FormulationAI) for *in silico* formulation design. First, the most comprehensive datasets of six widely used drug formulation systems in the pharmaceutical industry were collected over 10 years, including cyclodextrin formulation, solid dispersion, phospholipid complex, nanocrystals, self-emulsifying and liposome systems. Then, intelligent prediction and evaluation of 16 important properties from the six systems were investigated and implemented by systematic study and comparison of different AI algorithms and molecular representations. Finally, an efficient prediction platform was established and validated, which enables the formulation design just by inputting basic information of drugs and excipients. FormulationAI is the first freely available comprehensive web-based platform, which provides a powerful solution to assist the formulation design in pharmaceutical industry. It is available at https://formulationai.computpharm.org/.

## INTRODUCTION

With the rapid development of science and technology, the pharmaceutical industry faces great pressure to employ more efficient and systematic ways in the drug discovery and development process [1, 2]. In the drug discovery area, there are various widely used techniques to accelerate different stages of the whole process, such as high-throughput screening [3], combinatorial chemistry [4], computer-aided drug design (CADD) and artificial intelligence-driven drug design (AIDD) [5–8]. However, conventional formulation studies still strongly rely on personal experiences by trial-and-error experiments, resulting in a labor-consuming, tedious and costly pipeline [9]. These point-to-point experimental approaches restrict research and development efficiency, as they cannot predict the effectiveness of multiple formulations in advance. Moreover, experimental guidance based on personal experiences often leads to a lack of innovation and a tendency to stick to familiar methods. Consequently, this hinders the success rate of drug formulation research. Thus, it is highly required to develop intelligent and efficient methods for formulation development to keep pace with the progress of the pharmaceutical industry.

In recent decades, a new branch ‘computational pharmaceutics’ utilizes computational methods [e.g. artificial intelligence (AI), big data and multi-scale modeling techniques] to design, optimize and evaluate the formulation of drugs [10–12]. AI algorithms are able to analyze large amounts of data generated from experiments or simulations to extract meaningful insights into the behavior of formulations [9, 10, 13, 14]. The integration of AI technology into pharmaceutics has the potential to revolutionize the field of pharmaceutical research by enabling faster, more efficient and more accurate drug formulation and evaluation [15]. There is no doubt that these AI prediction models have played an important role in the design and evaluation of some drug formulations. They can not only predict not only the basic physicochemical properties of active pharmaceutical ingredients (APIs) but also the key parameters of the formulation systems by searching high-dimensional formulation space with the drug/excipients/process combination [16, 17]. Furthermore, the algorithms can analyze the influencing factors to theoretically guide the formulation design [10].

However, successfully applying these models still faces a series of challenges [18–20]. First, model reproducibility often comes with certain difficulties due to different operating environments. Second, in general, pharmaceutical scientists have a professional gap in programming, which creates inherent barriers to applying these models. Of course, the conservatism of industrial data also exacerbates the problem of model sharing [21]. Therefore, a unified framework/computational platform to translate the models into directly applicable tools is highly needed.

**Figure 1:**
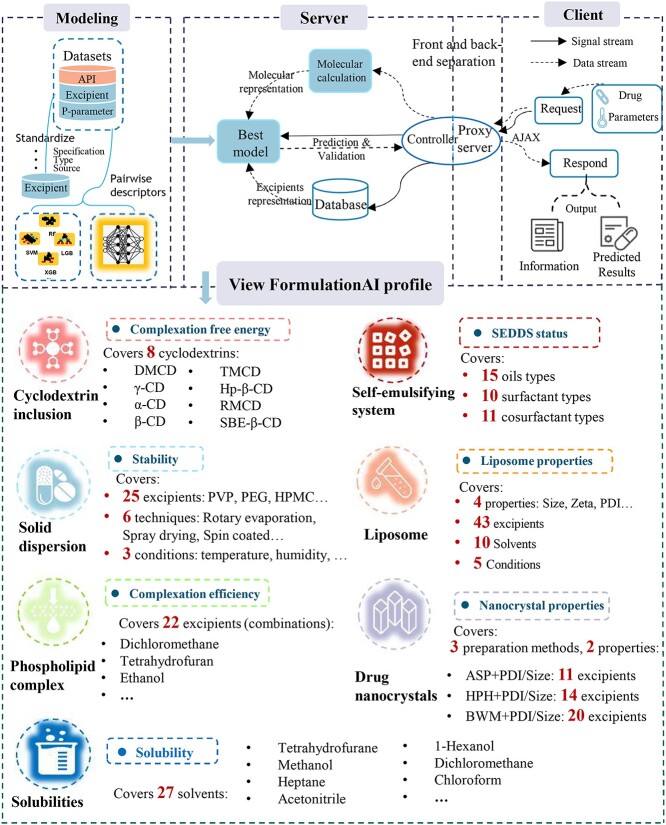
The workflow of FormulationAI. The implementation of this platform included several stages. First, the raw data were collected and cleaned manually. The specifications, sources and structures were standardized. Second, new representation strategies were conducted for the six delivery systems using pairwise descriptors; different representative algorithms and feature selection methods were utilized to build and selected accurate and efficient models for webserver deployment. Third, an easy-to-use web platform was established based on a modular computational framework to guarantee optimal efficiency and intelligent calculating process. This workflow displayed the implementation process and the main functionalities covered by FormulationAI.

This paper presents the construction of the first comprehensive AI computational platform ‘FormulationAI’ through systematic data mining and AI modeling analysis (Figure 1). In the first stage, the focus is on solving the problem of solubility enhancement for poorly soluble drugs, covering the evaluation of 16 important properties in six systems, such as cyclodextrin (CD) formulation, solid dispersion (SD), nanocrystal, self-emulsifying drug delivery system (SEDDS), liposome and various solvent systems. One of the most notable features of this platform is its ability to make fast predictions without the need for any complex theoretical calculations or wet lab experiments. This is made possible by the platform’s built-in procedures, which unify descriptors and inputs, allowing users to simply submit a drug structure and receive a quick prediction. For pharmaceutical scientists, FormulationAI provides new ideas and technical means to address challenges in drug delivery. It greatly accelerates the efficiency of discovering and evaluating new formulations. For the industry, it will break the limitations of relying mainly on traditional wet experiments for formulation evaluation by providing a completely new option for them. Furthermore, it holds significant value in enhancing and improving existing formulation systems in their product pipelines. To sum up, the proposed platform will significantly facilitate drug product development by reducing the timeline and saving materials.

## MATERIAL AND METHODS

### Data set construction and pretreatment

In the first stage of the FormulationAI research process, we started with the widely used six drug delivery systems. In addition, the solubility of drug molecules in different solvents was systematically studied. In the process of data collection, relevant data were collected from public databases and published literature over 10 years (each data set is slightly different), combined with some internal data to form 15 property data sets and 1 solubility data set for the six delivery systems.

#### Data collection

**Figure 2:**
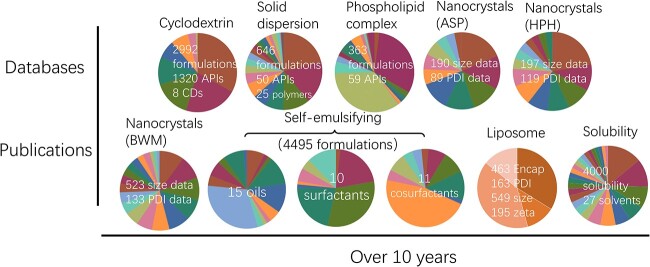
The overview of the data sets in FormulationAI. Data sets of six drug delivery systems and solubility were displayed in pie graphs. Different colors represent the proportions of different excipients/solvents in the data items. Colors in the pie of the liposome system represent the proportions of endpoints. The colors are independent for each pie, and the detailed types of data can be found in the ‘documentation’ section of the platform.

CD as a commonly used excipient in pharmaceutical products was first considered in this project. For this data set, 2992 formulation data records from 1980 to 2018 were collected, including 1320 APIs and eight types of CDs. The complexation-free energy (ΔG) of host–guest complexation was used as the output in the model [22]. For SD formulation, good physical stability is a basic requirement for a qualified design [23]. Here, 646 physical stability data items were collected and refined from the literature, which included 50 drugs and 25 kinds of polymers. In the physical stability test for SDs, at least 3–6 months were required. Herein, the physical stability of 3 and 6 months were treated as two endpoints for prediction separately [24]. For the phospholipid complex, 363 formulation data with 59 APIs were collected from literature ranging from 1999 to 2019. Eighty percent of the complexation rate (binary complex system) was considered as the threshold value [25, 26]. Nanocrystals have exhibited great advantages in solving the dissolution issue of water-insoluble drugs [27, 28]. The size and polydispersity index (PDI) were two important properties of drug nanocrystals. Here, data sets for three nanocrystal preparation methods were prepared [29]. For the anti-solvent method (ASP), 190 size data and 89 PDI data were collected; for the high-pressure homogenization (HPH) method, 197 size data and 119 PDI data were collected; and for the ball wet milling (BWM) method, 523 size data and 133 PDI data were collected [29]. The SEDDS, a thermodynamic stable nano formulation, consists of oil, surfactant, co-surfactant and APIs [30]. Here, a pseudo-ternary phase system was studied. A data set of 4495 formulation samples including 15 types of oils, 10 types of surfactants and 11 types of cosurfactants was prepared [31]. Self-emulsion status of the formulation under specified conditions was to be predicted [31]. Liposome is regarded as a promising drug delivery system due to its great biocompatibility, biodegradability and low toxicity [32]. The composition of lipids, particle size, surface charge and lamellarity would decide the structure and morphology of liposome particles. Herein, 463 encapsulation data, 163 PDI data, 549 size data and 195 zeta potential data were collected for modeling. The solubility of drugs in different solvents is crucial for drug formulation design. Here, we collected the current largest data set containing more than 4000 solubility points in 27 solvents [33]. The overview and statistical information of the above-mentioned data set are displayed in Figure 2. The definition and description of the above properties could be found on the ‘Documentation’ section on the website.

#### Data pretreatment

Different data sets undergo different preprocessing steps and methods, and the detailed process can refer to the corresponding published literature. During the construction of the webserver, the data accuracy, experimental conditions and classification criteria are consistent with the corresponding paper. However, due to the use of slightly different molecular recognition and representation methods, and different backend processing programs, there may be slight deviations in the data included. Consequently, the results of the model may not be exactly the same as previous publications. This does not affect the accuracy of the built-in models and published models in FormulationAI.

### Molecular representation

The formulation is always formed by more than two components including drugs and excipients. In some cases, the excipients even could be a mixture [31]. Thus, current difficulties are how to properly characterize the formulation with molecular descriptors. In this study, the general small molecular descriptors were used to represent drugs, while the combination of both physicochemical descriptors and functional parameters of excipients was employed to characterize excipients. When building models, we utilized different transformation strategies to construct drug–excipient pairwise descriptors, which have also been partly successfully applied in our previous publications [22, 24, 26, 29, 31, 33].

**Figure 3:**
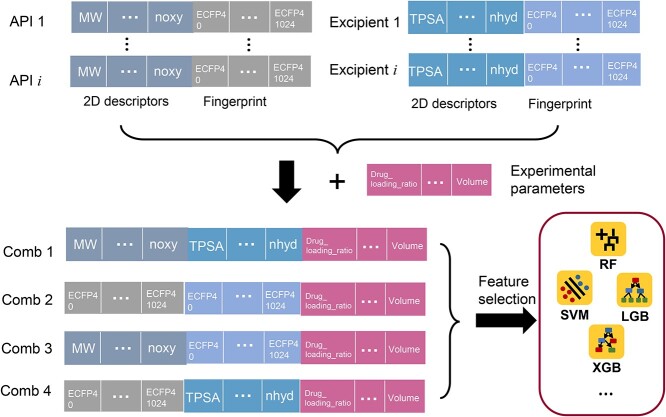
The molecular representation strategy of FormulationAI. Molecular descriptors and fingerprints of drug molecules and excipients were first calculated separately and then combined with experimental parameters. The advanced AI algorithms were then compared and selected to build robust models. The API is short for ‘active pharmaceutical ingredient’. The ‘2D descriptors’ means ‘2-dimensional descriptors’. ‘Comb 1’ is short for ‘combination 1’. The RF, SVM, LGB and XGB are short for random forest, support vector machine, LightGBM and XGBoost.

As shown in Figure 3, ChemDes [34] and PyBioMed [35] were used as descriptor calculation software for the molecular description of drugs (APIs). A total of 775 2D molecular descriptors and seven commonly used fingerprints were selected, and the specific categories and numbers can be seen in Table S1. For excipients, we first classified and unified the types, grades and molecular structures of excipients in different dosage forms. For example, in the SD delivery system, PEGs used were categorized as PEG1500, PEG2000, PEG4000, PEG6000, PEG8000 and PEG9000 [24]. The specific categories can be found in the ‘Documentation’ section of the website. The molecular characterization of excipients was divided into two parts. One part was the basic parameters of the classified excipients, including monomer molecular weight and polymerization degree. The other part was the small molecular descriptors of the monomers, which were consistent with the descriptors of APIs. Finally, we built drug–excipient pairwise descriptors. The method of constructing pairwise descriptors adopted the built-in method from PyBioMed. The strategy of direct splicing can balance the computation speed and accuracy. The construction process of pairwise descriptors is shown in Figure 3, which consisted of three parts: API features, excipient features, and process parameters. We selected one of the four combinations based on different tasks.

### Algorithms for AI modeling

In this project, the modeling process was divided into three steps. The prepared data sets were first fed into the modeling pipeline of Pycaret (https://pycaret.org/) in Python (3.7) environment and KNIME software (https://www.knime.com/). Models with different algorithms (over 10) will be compared and selected. In the third step, based on the results from the second step, we will further optimize the parameters and perform feature selection and finally, obtain the best model. Typically, the best model often comes from the following algorithms (Figure 3), including random forest (RF) [36], support vector machine (SVM) [37], LightGBM [38] and XGBoost [39]. SVM as a well-established and proven algorithm has a strong theoretical foundation, which can handle non-linearly separable data. RF can handle high-dimensional and complex data, including both categorical and numerical data. It is highly interpretable and easy to understand and visualize. LightGBM and XGBoost are fast and efficient, especially when working with large data sets. For problems in computational pharmaceutics, these algorithms have shown greater advantages than deep learning due to relatively small data and feature quantities, which were discussed in previous publications [22, 24, 29, 33].

In terms of feature selection, we proceed in two steps. First, for continuous numerical descriptors, irrelevant variables were removed with a variance less than 0.05; one of any two variables with a correlation greater than 0.95 (0.99 for some cases) was removed. The experimental conditions and process parameters were kept because these parameters are clearly known to influence the formulation design. Second, recursive feature elimination is used to select the remaining descriptors, resulting in the best subset of descriptor combinations.

### Performance evaluation

The data sets were split into training and test sets with 5-fold or 10-fold cross-validation on the training set. For regression models, we mainly evaluate the cross-validation coefficient of determination (*Q*^2^), mean absolute error (MAE) and root mean squared error (RMSE), as well as the test set’s coefficient of determination (*R*^2^), MAE and RMSE. For classification models, we evaluate the cross-validation and test set’s accuracy (ACC), area under the receiver operating characteristic curve (AUC), sensitivity (SE) and sensitivity (SP), respectively.

### Implementation

The platform was designed to provide a high-performance computational environment for multi-user real-time computing. To achieve this, we utilized Alibaba Cloud’s Elastic Compute Service (ECS) as the underlying hardware platform and selected Ubuntu as the server operating system. To handle web requests and resource access, uWSGI (https://pypi.org/project/uWSGI/) and Nginx (https://www.nginx.com/) were employed, as the combination of these two technologies allows for load balancing and security. The platform was developed using the Python programming language, which is popular in the programming community due to its mature community and availability of AI and data processing packages like Numpy (https://numpy.org/), Pandas (https://pandas.pydata.org/) and Scikit-learn (https://scikit-learn.org/stable/). The framework of the platform was established using Django (https://www.djangoproject.com/), which provides a clear separation of business data (models) from user interfaces (views) and makes it easier to upgrade and maintain the platform. The data were stored using the widely used relational database, MySQL (https://www.mysql.com/). On the front-side, we unitized asynchronous JavaScript and XML (AJAX) for asynchronous data retrieval, CSS (cascading style sheets) and JavaScript (https://www.javascript.com/) for building a cross-platform user interface and Transport Layer Security (TLS) protocol for data transmission security. With these technologies in place, FormulationAI was able to successfully store and format the evaluated models in the established framework so as to provide efficient prediction service.

## RESULTS

### Model performance

**Figure 4:**
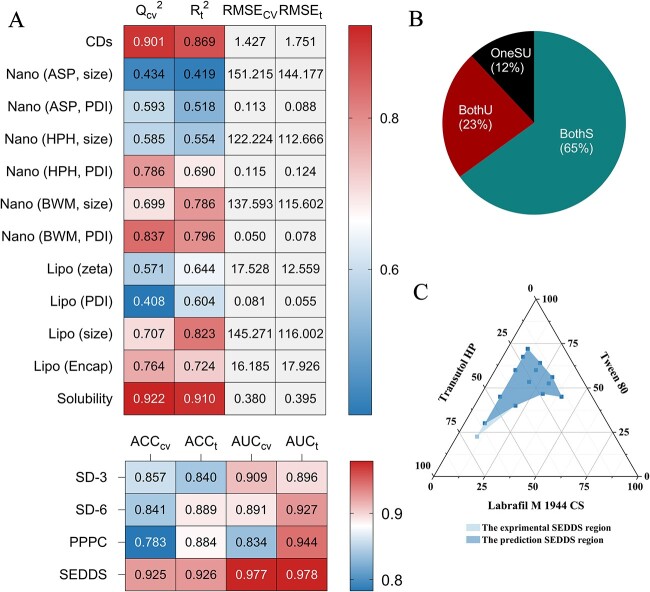
Statistical summary of the model performance and validations of the selected best models. The performance of all the best models. (**A**) The best results of the 12 regression models and four classification models. Comparable indicators were displayed as heat maps, and the color depth represents the value. (**B**) The proportion of predicted SD stabilities (3 and 6 months) compared with experimental results for marketed formulations. ‘BothS’ represents the marketed formulations that were predicted as stable both in 3 and 6 months. ‘BothU’ represents both unstable. ‘OneSU’ represents just unstable in 6 months. (**C**) The three-phase diagram of the example SEDDS formulation. The experimental and predicted regions were rendered in different colors.

The results of the 16 best models (12 regression models and 4 classification models) are summarized in Figure 4A. The detailed results of each model can be found in Tables S2 and S3 and Figures S1–S16. The optimized parameters and selected features of the best models are listed in Tables S4 and S5. Overall, the performance of the models was satisfactory. For regression tasks, the best-performing model was the solubility prediction model of drugs in different solvents, and the LightGBM and RF algorithms performed best on this data set, with *Q*^2^ of 0.922 and 0.910 for cross-validation and test set, respectively. The prediction of the complexation free energy (ΔG) of CD also performed well with *R*^2^ of 0.869 for the test set and 0.901 for the cross-validation. Most of the nanocrystal and liposome prediction models performed well within an acceptable range, but the accuracy of the size prediction for the three nanocrystal preparation methods was not so good. This is partly due to the limited amount of data and the addition of stabilizers, which makes binary pairwise descriptors become ternary descriptors, diluting the information in the training space. In addition, the proportion of data from different excipients can also significantly affect the accuracy of the model. When the amount of data for some commonly used excipients is significantly higher than that of others, the accuracy of the model may decrease. For example, the performance of the two BWM models was better than their corresponding models in ASP and HPH because the proportion of excipients in the BWM data set was more balanced than another two methods. For classification tasks, all four models performed well, with test set ACC exceeding 0.8 and AUC exceeding 0.9. The results of cross-validation were also consistent with those of the test set.

FormulationAI includes 16 models that address 16 important formulation properties across six different delivery systems. It is worth noting that, to our knowledge, no similar predictive models were reported to solve the same problems except the methodology papers we previously published. Take the commonly used SDs as an example. There have been some computational methods to study the properties of SDs. However, most of these studies focus on specific drugs or specific excipients, which means the modeling or prediction has not been carried out based on a large scale of data. For example, as early as 1989, Bonelli *et al*. [40] studied the relationship between the release rate of erythromycin and a series of physicochemical properties of polymers, using simple linear algorithms. Aleksander *et al*. created a simple artificial neural network model for the dissolution characteristics of ketoprofen SDs in 2005 [41]. Later, there have been some studies predicting the behavior of SDs in specific excipient systems [42, 43]. Although previous studies have provided effective guidance for the rational construction of SD formulations with specific drugs or polymers, they often lacked generality. In contrast, we have developed a universal prediction model for the 3- and 6-month stability of SDs. Although we cannot directly compare the performance of our model to these prior works, it is clear that FormulationAI provides a superior solution in terms of problem importance, data dimensionality, applicability and computational efficiency.

During the model building process, we overcame a series of obstacles. First, one of our challenges was how to properly characterize excipients. Excipients are typically large and flexible molecules that possess complex structures and functions, usually with repeat units or artificial modifications. To address this issue, we created a new methodology to standardize them, including their specification, model, source and modification types. From this basis, we determined the structure of each monomer and repeat unit, ensuring that the excipient descriptors effectively and accurately capture their physicochemical properties. Second is the accessibility of descriptors. It is essential to ensure that the descriptors used can be computed on the server-side, and thus descriptors from commercial software and non-deployable software in our previous publications were not included in this project. For example, in the CD system, we have tested a series of descriptors, including those calculated by ALOGPS 2.1 and ChemAxon software (https://chemaxon.com/) [7]. These descriptors can also be used to build models with acceptable performance, but either they are not available for free or there is no direct program interface available. Third is the operability of the selected features. The optimal subset of selected features needed to be manually adjusted to balance the inputs during platform construction and the actual meaning of descriptors. Fourth is the practicality of model selection. Among the algorithms with similar performance, we selected ones that were more robust, repeatable and easier to deploy. Actually, these two aspects are interrelated. We must comprehensively consider the performance of the model, deployment requirements, computational speed and other requirements, so the models were all rebuilt, which aims to convert drug formulation issues into a reasonable and intelligent calculation process. For example, some experimental condition parameters were kept, although they may not have high feature importance but have important practical value. During the process of selecting the optimal subset, we also systematically compared the impact of different algorithms and descriptors on model performance. For example, in the construction of six models for nanocrystals, we adopted the unified pairwise description strategy for descriptor selection and feature engineering. After comprehensive consideration of the above factors, we obtained better models than the ones from He *et al*. [10]. As an example, in the size prediction model of the ASP data set, our best model achieved an MAE of 100.3 and 94.6 in 10-fold cross-validation and the test set, respectively, whereas their model had an MAE of 111.7 and 120.7. Similarly, in the PDI prediction model of the ASP data set, our best model achieved an MAE of 0.061 and 0.054 in 10-fold cross-validation and the test set, respectively, while their model had an MAE of 0.105 and 0.086. Similarly, the performance of the other four models was also significantly improved. Naturally, there are also some other cases. For example, in the prediction of the complexation rate of the phospholipid complex, we utilized a larger data set than what was used in the work of Gao *et al*. [9]. But their work included experimental properties such as LogS, melting temperature of drugs, boiling point and vapor pressure of the solvents. These properties cannot be directly calculated from the structure on the web server. Therefore, the performance of the selected model was slightly reduced compared to their model, but its practical value was greatly improved. In summary, addressing these problems made the computational models move from theoretical results to practical applications.

### Webserver

**Figure 5:**
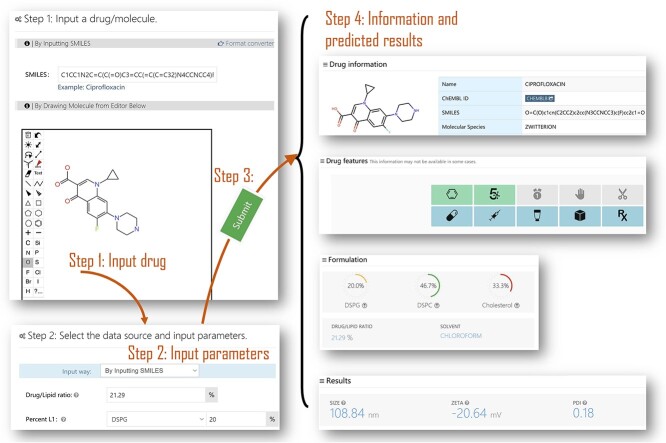
A snapshot of the user interface using ciprofloxacin as an example.

The user interface of the platform includes several modules: ‘Homepage’, ‘Webserver’, ‘Documentation’, ‘Help’ and ‘Publications’. The ‘Webserver’ serves as the entrance for all calculation modules; the ‘Documentation’ module describes information related to these delivery systems; the ‘Help’ module gives a short video to assist users in quickly getting started; and the ‘Publication’ module lists relevant literature for further study. Figure 5 displays the overview of the user interface and the snapshot of processing an example.

#### Input

To start the calculation, users are first required to select the corresponding module and input the SMILES [44] of the drug and the corresponding parameters. Users also can draw a molecular structure to proceed. FormulationAI is an intelligent processing system that accomplishes all related analysis steps automatically.

#### Output

At present, 16 properties mentioned above have been implemented. Output/results were mainly presentations for regression and classification tasks. (1) For the classification task, we will give the predicted label and probabilities of each category in the form of rich text or tables. (2) For the regression task, we will report the actual predicted values of the endpoint. (3) On the result page, three sections were designed to show the basic information about the drug structures and excipients, the process/experimental conditions and the prediction results. (4) All the results were rendered by different rich text styles or interactive charts for a better understanding.

### Case study and validation

#### Case study 1

In the prediction of SD stability, an example of particular interest comes from estradiol and PVP K30. Figure S17 shows the prediction results by filling different drug loading ratios (1:2, 1:5); the predicted results revealed that the 1:2 ratio exhibited poor stability with stable probabilities of 0.51 (3-month) and 0.38 (6-month), while the 1:5 ratio showed comparatively good stability with probabilities of 0.63 (3-month) and 0.55 (6-month). This result was validated in real experiments, which showed that the 1:5 ratio was stable under the 6-month accelerated conditions, while the 1:2 was not [24]. We also compared the experimental data of the marketed formulations with the prediction results of FormulationAI and found that more than 65% of the stability results met expectations (Figure 4B).

#### Case study 2

The SEDDS formulation using the combination of Labrafil M 1944 CS, Tween 80 and Transcutol HP as the oil, surfactant and cosurfactant was selected as another example. First, 20, 64 and 16% were inputted as the oil, surfactant and cosurfactant proportions, which were determined by mixing the surfactant and cosurfactant in a mass ratio of 4:1 to form a mixed emulsifier, and then oil was added to the mixed emulsifier at a mass ratio of 2:8; the process parameters were set to pH = 7.0 and concentration A = 1%. The predicted status indicated a success (Lable: +) (Figure S18). Then, the parameters were adjusted to generate predicting results of six ratios of surfactant and cosurfactant (4:1, 3:1, 2:1, 1:1, 1:2 and 1:3) and the nine mixing ratios of oil and emulsifier (1:9, 2:8, 3:7, 4:6, 5:5, 6:4, 7:3, 8:2 and 9:1) [31]. Finally, a total of 54 different combinations are obtained to plot the three-phase diagram (Figure 4C). The result indicated that the predicted values were in good agreement with experimental data.

### Brief discussion and perspective

The above examples demonstrate the accuracy and practicality of FormulationAI in specific drug delivery systems. Choosing the right delivery system is crucial in drug formulation design. The delivery system must be able to effectively deliver the drug to its intended target while minimizing potential side effects. In practical drug formulation design, we need to choose the appropriate delivery system for predictive evaluation based on the drug indication and mechanism, *in vivo* behavior and the physicochemical properties of drug molecules. In addition to predicting the effectiveness of delivery systems, FormulationAI can also be used to optimize existing formulations. By analyzing the properties of the drug molecule and the delivery system, FormulationAI can identify potential formulation space for improvement and suggest modifications that can enhance the efficacy and safety of the drug. Another promising application of FormulationAI is searching for appropriate formulations in case of drug repurposing, a field of increasing interest, because an existing drug formulation could be not suitable for a different repurpose of the same drug. By leveraging FormulationAI, researchers can efficiently explore and identify formulations that can be repurposed for new therapeutic uses, potentially accelerating the drug development process and expanding treatment options for various diseases and conditions.

Despite powerful functionalities, certain points should be noted for the correct utilization of FormulationAI in practical applications. First, it is crucial to pay attention to the relationship between input conditions and real experimental conditions, especially ensuring correct data units and percentage units. Second, the drug APIs used in this project are single-component compounds, so it is necessary to use prototype drugs during the experimental process. If other forms of drug materials are used, it is essential to exclude the interference of solvents and other components. Furthermore, the platform has imposed limitations on experimental conditions and parameter ranges, based on the credible range of various indicators of the current model, which is of reference significance. In experimental processes, some conditions may exceed the restricted range of the platform, and researchers should make appropriate decisions accordingly. Of course, the current version also has some limitations. On the data level, although the current model employs the largest data set available so far, there is still insufficient coverage for some delivery systems, especially those involving multiple components. Better data contribution schemes from pharmaceutical community or industry may overcome this barrier. There is still room for improvement in the accuracy of the models for certain delivery systems, such as nanocrystals. In future maintenance and updates, we will strive to enhance and improve the methods based on the situation. Additionally, as technology advances, new delivery systems will gradually be incorporated into the FormulationAI platform. We believe that, as work advances further and deeper, FormulationAI will provide more value to the pharmaceutical community and industry.

## CONCLUSION

We successfully constructed the first comprehensive AI computational platform ‘FormulationAI’ for *in silico* drug formulation design. By utilizing new strategies for drug–excipient pairwise representation and different AI algorithms, robust models of 16 important properties for six systems in the current plan have been established. After addressing several technical difficulties, the model has been successfully deployed on high-performance computing servers with an easy-to-use interface. FormulationAI built a bridge between pharmaceutics and computer science, which is able to shift the paradigm of conventional formulation development procedures from experience-dependent studies to data-driven methodologies. Furthermore, it made a great step forward in transitioning AI models from theory to application in computational pharmaceutics. During the beta testing period, hundreds of institutions in the pharmaceutical industry and academia worldwide have used our server and provided very positive feedback, which reflects the scientific and practical value of FormulationAI. In the future, we are striving to improve the efficiency of the calculation process and establish sorting and scoring schemes for different outputs. We will keep updating the platform with more predictive models and formulation data. The study and evaluation of important properties of other delivery systems are also in our plan. We strongly believe that ‘FormulationAI’ will become an essential tool for pharmaceutical scientists both in academia and industry.

**Key Points**

1. Constructed the first comprehensive AI-based platform for *in silico* drug formulation design.
2. Established the largest data sets and robust AI models for six important drug delivery systems.
3. Provided an easy-to-use and fast prediction interface transitioning AI models from theory to application.

## Supplementary Material

sp20231010_bbad419

## Data Availability

FormulationAI is freely accessible to users at https://formulationai.computpharm.org. Extensive documentation that explains all the data sets and models is available at https://formulationai.computpharm.org/home/doc.

## Notes

**Jie Dong** is an associate professor of Central South University. His research interests include artificial intelligent systems for drug discovery, the development of software/web servers/databases in chem/bioinformatics and drug discovery.

**Zheng Wu** is currently a postgraduate student of Medical Administration (MAD) at the University of Macau, working in the field of computational pharmaceuticals.

**Huanle Xu** is an assistant professor of University of Macau. His research focuses on machine learning algorithms and their practical applications in addressing real-world challenges.

**Defang Ouyang** is an associate professor of University of Macau. His research focused on computational pharmaceutics including artificial intelligence of pharmaceutical formulations, multi-scale modeling in drug delivery and pharmacoinformatics.
