# Supplementary material for: FormulationAI: a novel web-based platform for drug formulation design driven by artificial intelligence
Source: Brief Bioinform. 2023 Nov 22;25(1):bbad419. doi: 10.1093/bib/bbad419 (PMC10783856; doi:10.1093/bib/bbad419)
Supplement: sp20231010_bbad419 [file sp20231010_bbad419.pdf]

FormulationAI: a novel web-based platform for drug formulation design driven by artificial intelligence

Jie Dong[1, 2], Zheng Wu[2], Huanle Xu[3], Defang Ouyang[2, *]

[1]Xiangya School of Pharmaceutical Sciences, Central South University, Changsha, China

[2]Institute of Chinese Medical Sciences (ICMS), State Key Laboratory of Quality Research in Chinese Medicine, University of Macau, Macau, China

[3]Faculty of Science and Technology, University of Macau, Macau, China

*Corresponding author: Defang Ouyang, +853-88224514, defangouyang@um.edu.mo

Supplementary Material

Table S1. Small molecular descriptors and fingerprints used in the construction of drug-excipient pairwise descriptors

Descriptors	Number/length
Constitution	30
Connectivity descriptors	44
Basak descriptors	21
Burden descriptors	64
Topology descriptors	35
Kappa descriptors	7
E-state descriptors	237
Moran autocorrelation descriptors	32
Geary autocorrelation descriptors	32
Molecular property descriptors	6
Moreau-Broto autocorrelation descriptors	32
Charge descriptors	25
MOE-type descriptors	60
CATS2D descriptors	150
Daylight-type fingerprints	2048
MACCS fingerprints	166
Atom pairs fingerprints	2048
TopologicalTorsion fingerprints	2048
E-state fingerprints	79
ECFP4 fingerprints	1024
PubChem fingerprints	881

Table S2. The model performance of best selected models (regression)

	Algorithm	Cross-validation			Test set		
		Q^2	MAE	RMSE	R^2	MAE	RMSE
CDs	LightGBM	0.901	1.12	1.427	0.869	1.348	1.751
Nano (ASP, size)	RF	0.434	100.289	151.215	0.419	94.563	144.177
Nano (ASP, PDI)	RF	0.593	0.061	0.113	0.518	0.054	0.088
Nano (HPH, size)	RF	0.585	82.938	122.224	0.554	76.791	112.666
Nano (HPH, PDI)	RF	0.786	0.0767	0.115	0.690	0.0751	0.124
Nano (BWM, size)	RF	0.699	81.997	137.593	0.786	58.533	115.602
Nano (BWM, PDI)	RF	0.837	0.020	0.050	0.796	0.027	0.078
Lipo (Zeta)	RF	0.571	11.086	17.528	0.644	8.299	12.559
Lipo (PDI)	RF	0.408	0.059	0.081	0.604	0.042	0.055
Lipo (Size)	RF	0.707	75.263	145.271	0.823	67.198	116.002
Lipo (Encap)	RF	0.764	10.853	16.185	0.724	11.333	17.926
Solubility	RF	0.922	0.180	0.380	0.910	0.177	0.395

Table S3. The model performance of best selected models (classification)

	Algorithm	Cross-validation				Test set			
		ACC	AUC	SE	SP	ACC	AUC	SE	SP
SD-3	RF	0.857	0.909	0.924	0.714	0.840	0.896	0.964	0.577
SD-6	RF	0.841	0.891	0.901	0.740	0.889	0.927	0.961	0.763
PPPC	RF	0.783	0.834	0.894	0.570	0.884	0.944	0.925	0.828
SEDDS	RF	0.925	0.977	0.922	0.927	0.926	0.978	0.927	0.926

Table S4. The selected best algorithms and parameters for the best models

Model	Algorithm	Best parameters
CDs	LightGBM	'boosting_type': gbdt,<br>'learning_rate': 0.06,<br>'n_estimators': 531,<br>'num_leaves': 55,<br>'max_depth': 15
Nano (ASP, size)	RF	'max_features': 1, 'n_estimators': 500,<br>'oob_score': True, 'cv': 10
Nano (ASP, PDI)	RF	'max_features': 28, 'n_estimators': 500,<br>'oob_score': True, 'cv': 10
Nano (HPH, size)	RF	'max_features': 37, 'n_estimators': 500,<br>'oob_score': True, 'cv': 10

Nano (HPH, PDI)	RF	'max_features': 33, 'n_estimators': 500, 'oob_score': True, 'cv': 10
Nano (BWM, size)	RF	'max_features': 11, 'n_estimators': 500, 'oob_score': True, 'cv': 10
Nano (BWM, PDI)	RF	'max_features': 7, 'n_estimators': 500, 'oob_score': True, 'cv': 10
Lipo (Zeta)	RF	'max_features': 16, 'n_estimators': 500, 'oob_score': True, 'cv': 10
Lipo (PDI)	RF	'max_features': 1, 'n_estimators': 500, 'oob_score': True, 'cv': 10
Lipo (Size)	RF	'max_features': 25, 'n_estimators': 500, 'oob_score': True, 'cv': 10
Lipo (Encap)	RF	'max_features': 30, 'n_estimators': 500, 'oob_score': True, 'cv': 10
Solubility	RF	'max_features': 12, 'n_estimators': 500, 'oob_score': True, 'cv': 10
SD-3	RF	'max_features': 6, 'n_estimators': 500, 'oob_score': True, 'cv': 5
SD-6	RF	'max_features': 19, 'n_estimators': 500, 'oob_score': True, 'cv': 5
PPPC	RF	'max_features': 15 'n_estimators': 500, 'oob_score': True, 'cv': 10
SEDDS	RF	'max_features': 8, 'n_estimators': 500, 'oob_score': True, 'cv': 5
Note: the names and definitions of the parameters can be found at: https://scikit-learn.org/0.18/		

Table S5. The selected descriptors that were used in the best models

Model	Selected descriptors
CDs	
Nano (ASP, size)	Concentration(mg/ml)_API,MW_Stabilizer1,MolecularWeight_Stabilizer1,Concentration(mg/ml)_Stabilizer1,VolumeofWater(ml),UltrasonicationPowerInput(W),UltrasonicationTime(min),Vorganic/Vwater,ATSe4,ATSm7,AWeight,CATS_AL9,CATS_DL4,CATS_DL9,CATS_LL9,CIC2,GATSe1,MATSm1,Solvent_Qass,Solvent_VSAEstate8,Solvent_bcutp9,Stabilizer1_CATS_AL6,Stabilizer1_CATS_AL9,Stabilizer1_CATS_DD0,Stabilizer1_CIC2,Stabilizer1_CIC6,Stabilizer1_Chi0,Stabilizer1_GATSm4,Stabilizer1_GATSv1,Stabilizer1_IC0
Nano (ASP, PDI)	ATSe3, ATSe4, CATS_AA4, CATS_AL3, CATS_LL4, Concentration(mg/ml)_API, Concentration(mg/ml)_Stabilizer1, MW_Stabilizer1, Solvent_QCmin, Solvent_SIC0, Solvent_bcutp11, Solvent_bcutp9, Solvent_slogPVSA1, Stabilizer1_AWeight, Stabilizer1_CATS_AA0, Stabilizer1_CATS_AL2, Stabilizer1_CATS_AL6, Stabilizer1_CATS_DD0, Stabilizer1_CIC0, Stabilizer1_CIC2, Stabilizer1_Chi4pc, Stabilizer1_GATSm3, Stabilizer1_GATSm4, Stabilizer1_GATSp2, StirringSpeed(r/min, UltrasonicationPowerInput(W), UltrasonicationTime(min), VolumeofWater(ml), Vorganic/Vwater, bcutp9
Nano (HPH, Size)	AW,CATS_AA0, CATS_AA2, CATS_AA4, CATS_AL4, CATS_DL3, CATS_DL4, CATS_LL6, CATS_LL8, Chi5, Concentration(mg/ml)_API, Concentration(mg/ml)_Stabilizer1, CycleIndex, DZ, GATSe3, GATSe5, GATSe6, GATSv7, MW_Stabilizer1, MW_Stabilizer2, MolecularWeight_Stabilizer1, PEOEVSA13, Pressure(Bar), Stabilizer1_GATSm4, Stabilizer1_GATSp4, Stabilizer1_GATSv3, Stabilizer1_GATSv5, Stabilizer1_IC1, Stabilizer1_LogP2, Stabilizer1_Mpc,

	Stabilizer1_QHss, Stabilizer1_Smax35, Stabilizer1_bcute2, Stabilizer1_bcute3, Stabilizer1_bcutm12, Stabilizer1_bcutp2, Stabilizer1_dchi2, Stabilizer1_slogPVSA4, Stabilizer2_AWeight, Viscosity_Stabilizer1
Nano (HPH, PDI)	CATS_AA0, CATS_AA4, CATS_AL4, CATS_AL7, CATS_DA0, CATS_LL4, CIC0, CIC6, Chi6ch, Chiv10, Concentration(mg/ml)_API, Concentration(mg/ml)_Stabilizer1, Concentration_Stabilizer2, CycleIndex, GATSv6, MW_Stabilizer1, MolecularWeight_Stabilizer1, NofRepeatUnits_Stabilizer1, PEOEVSA10, Pressure(Bar), Stabilizer1_GATSm3, Stabilizer1_GATSp4, Stabilizer1_IC0, Stabilizer1_IC4, Stabilizer1_LDI, Stabilizer1_LogP, Stabilizer1_Mac, Stabilizer1_Mpc, Stabilizer1_QCmin, Stabilizer1_Rpc, Stabilizer1_SIC1, Stabilizer1_bcute11, Stabilizer1_bcutm3, Stabilizer1_bcutv1, Stabilizer1_mChi1, Stabilizer1_slogPVSA2, Stabilizer2_AWeight, Temperature, Viscosity_Stabilizer1, Viscosity_Stabilizer2
Nano (BWM, size)	CATS_AA2, CATS_DA8, Chi4pc, Concentration(mg/ml), DiameterofMillingBalls(mm), GATSp6, IC5, LDI, MW_Stabilizer1, Mac, MolecularWeight_Stabilizer1, NofRepeatUnits_Stabilizer2, PC5, PEOEVSA6, QHss, QOss, Qass, RotationSpeed(rpm), SIC3, Stabilizer1_CATS_AL2, Stabilizer1_CATS_AL6, Stabilizer1_GATSm1, Stabilizer1_GATSm4, Stabilizer1_GATSv7, Stabilizer1_LogP, Stabilizer1_Mac, Stabilizer1_PEOEVSA11, Stabilizer1_Qmin, Stabilizer1_kappa2, Stabilizer1_kappa3, Stabilizer1_slogPVSA4, Stabilizer2_GATSe1, Stabilizer2_GATSv1, Stabilizer2_LogP2, Stabilizer2_MRVSA5, Time(h), Viscosity_Stabilizer1, concentration(mg/ml)_Stabilizer1, concentration(mg/ml)_Stabilizer2, slogPVSA4
Nano (BWM, PDI)	Amount(mg), CATS_AA3, CATS_DA7, CATS_DL2, CATS_NL6, Chi2, Concentration(mg/ml), DiameterofMillingBalls(mm), GATSp3, GATSp8, MW_Stabilizer1, MillingMachine, MolecularWeight_Stabilizer1, RotationSpeed(rpm), Stabilizer1_EstateVSA8, Stabilizer1_GATSe1, Stabilizer1_GATSe4, Stabilizer1_GATSe6, Stabilizer1_GATSm1, Stabilizer1_GATSp3, Stabilizer1_GATSp4, Stabilizer1_GATSp8, Stabilizer1_IC2, Stabilizer1_Mpc, Stabilizer1_QCmin, Stabilizer1_kappam2, Stabilizer2_CATS_NL1, Time(h), Viscosity_Stabilizer1, concentration(mg/ml)_Stabilizer1
PPPC	BP3, CATS_AA4, CATS_AA5, CATS_AA6, CATS_AL3, CATS_AL7, CATS_DL4, CATS_LL0, CATS_LL2, CIC3, Complexity3, DC3, GATSe4, HeavyAtom_C3, J, MATSe4, MATSe6, MATSe7, MATSe8, MATSm7, MATSp1, MATSp7, MRVSA6, MW3, Mnc, Molar_Ratio2, PEOEVSA10, PEOEVSA11, PEOEVSA6, Polar_Index_3, Rnc, S35, SPP, Smin16, T3, TPSA3, Time, VP3, XlogP33, bcutm1
Lipo (Zeta)	D_L_ratio, MW_L1, HBDC_L1, TPSA_L1, Percent_L1, MW_L2, XLogP3_L2, HBAC_L2, Rot_L2, Percent_L2, MW_L3, XLogP3_L3, HBDC_L3, Percent_L3, P_time, Sonication, Filtration, Diameter, T, E_solvent, CATS_AL4, CATS_AL5, CATS_AL7, CATS_DL5, CATS_LL4, CATS_LL5, CATS_NL4, CIC3, CIC4, GATSe4, GATSp1, GATSp2, GATSp3, GATSp4, GATSp5, GATSv6, Hy, MTPSA, Mnc, PEOEVSA7, QHss, QOmax, QOmin, Qmin, SIC3, Smin15, UI, bcute1, bcutm4, bcutp1
Lipo (PDI)	D_L_ratio, MW_L1, Percent_L1, MW_L2, XLogP3_L2, HBDC_L2, HBAC_L2, Percent_L2, MW_L3, HBDC_L3, Percent_L3, P_time, Sonication, Filtration, Diameter, T, E_solvent, CATS_AL5, CATS_DL2, CATS_DL3, CATS_DL6, CATS_DL9, CATS_LL4, CIC2, GATSe6, GATSe7, GATSe8, GATSm2, GATSm5, GATSm7, GATSm8, GATSp6, GATSv1, GATSv3, GATSv4, GATSv7, Hy, MRVSA4, MRVSA5, PEOEVSA1, PEOEVSA6, PEOEVSA9, QCmax, QOss, Qmax, S35, Smax15, Smax16, bcute1, bcutm1
Lipo (Size)	D_L_ratio, MW_L1, HBAC_L1, TPSA_L1, Percent_L1, MW_L2, XLogP3_L2, HBAC_L2, Percent_L2, MW_L3, XLogP3_L3, Percent_L3, P_method, P_time, Sonication, Filtration, Diameter, T, BP_solvent, E_solvent, CATS_AL3, CATS_AL5, CATS_AP2, CATS_DA3, CATS_LL0, CATS_LL1, CATS_LL6, CATS_NL1, CIC3, GATSe2, GATSe5, GATSm1, GATSm8, GATSp1, GATSp5, GATSp6, GATSv1, GATSv4, GATSv5, GATSv6, GATSv7, IC1, MRVSA9, QOmax, Qass, Qmin, Rpc, SIC3, bcutm1, bcutp2
Lipo (Encap)	D_L_ratio, MW_L1, HBDC_L1, HBAC_L1, TPSA_L1, Percent_L1, MW_L2, XLogP3_L2, HBAC_L2, Percent_L2, MW_L3, XLogP3_L3, HBDC_L3, Percent_L3, P_time, Sonication, Filtration, Diameter, T, HAC_solvent, E_solvent, CATS_AA2, CATS_AA6, CATS_AL6, CATS_AN3, CATS_DA6, CATS_DL4, CATS_LL5, CATS_NL4, Chiv10, GATSe1, GATSe6, GATSe8, GATSm1, GATSm2, GATSm5, GATSv2, LDI, LogP2, MRVSA6, PEOEVSA7, QCss, QHss, QNmax, QNss, Rnc, SIC0, bcutm2, bcutp1, dchi2
Solubility	T, Drug_CATS_AA0, Drug_CATS_AL5, Drug_CATS_DA0, Drug_CATS_DD6, Drug_CATS_DL3, Drug_CATS_DL4, Drug_CATS_LL1, Drug_CATS_LL6, Drug_CIC2, Drug_CIC5, Drug_GATSe2, Drug_GATSe3, Drug_GATSe4, Drug_GATSe5, Drug_GATSe6, Drug_GATSe8, Drug_GATSm2, Drug_GATSm3, Drug_GATSm5, Drug_IC0, Drug_IC2, Drug_LogP, Drug_LogP2, Drug_PEOEVSA8, Drug_Qmax, Drug_Rnc, Drug_Rpc, Drug_S16, Drug_S36, Drug_SIC4, Drug_SIC5, Drug_Smax15, Drug_Tac, Drug_bcute1, Drug_bcutm1, Drug_bcutp1, Drug_slogPVSA0, Drug_slogPVSA4, Solvent_Chi2, Solvent_GATSe2, Solvent_GATSm2, Solvent_GATSp2, Solvent_GATSv1, Solvent_Hy, Solvent_IC0, Solvent_LogP, Solvent_MRVSA4, Solvent_Mnc, Solvent_PEOEVSA7, Solvent_Rpc, Solvent_SIC0, Solvent_SIC1, Solvent_Tac, Solvent_bcutm1, Solvent_bcutm4, Solvent_bcutm9, Solvent_kappam2, Solvent_kappam3, Solvent_slogPVSA1
SD-3	N_repeat2, MW_unit2, MP2, Viscosity2, HBAC2, Rot_Bond_Count2, Complexity2, TPSA2, Drug_load_ratio2, T2_1, RH2, Technique, T2_2, ATSe8, Arto, CATS_AL2, CATS_AL7, CIC5, Chiv3, MATSp8, MATSv5, S17, SIC1, SPP, Smax15, Smax34, Smin16, bcute12, bcute2, bcute9

SD-6	N_repeat2, MW_unit2, MP2, HBDC2, HBAC2, Rot_Bond_Count2, Complexity2, TPSA2, Drug_load_ratio2, T2_1, RH2, Technique, T2_2, CATS_AL4, CIC5, GATSe4, GATSv5, MATSe3, MATSm3, MATSm7, MATSp5, MATSv3, SIC4, Smin16, bcutm16, bcutp2
PPPC	Molar_Ratio2, MW3, XlogP33, TPSA3, HeavyAtom_C3, Complexity3, DC3, BP3, VP3, Polar_Index_3, T3, Time, CATS_AA4, CATS_AA5, CATS_AA6, CATS_AL3, CATS_AL7, CATS_DL4, CATS_LL0, CATS_LL2, CIC3, GATSe4, J, MATSe4, MATSe6, MATSe7, MATSe8, MATSm7, MATSp1, MATSp7, MRVSA6, Mnc, PEOEVSA10, PEOEVSA11, PEOEVSA6, Rnc, S35, SPP, Smin16, bcutm1
SEDDS	MW_E1, LogP_E1, BP_E1, MP_E1, Den_E1, Vis_E1, HLB_E1, FP_E1, ST_E1, SP_value_E1, Dose_E1, MW_E2, LogP_E2, MP_E2, Den_E2, Vis_E2, HLB_E2, FP_E2, SP_valueE2, Dose_E2, MW_E3, LogP_E3, BP_E3, MP_E3, Den_E3, Vis_E3, FP_E3, Dose_E3, PH, Concentration
Note: the name and definition of each descriptor can be found at: https://github.com/gadsbyfly/PyBioMed or http://www.scbdd.com/chemdes/list-descriptors/	

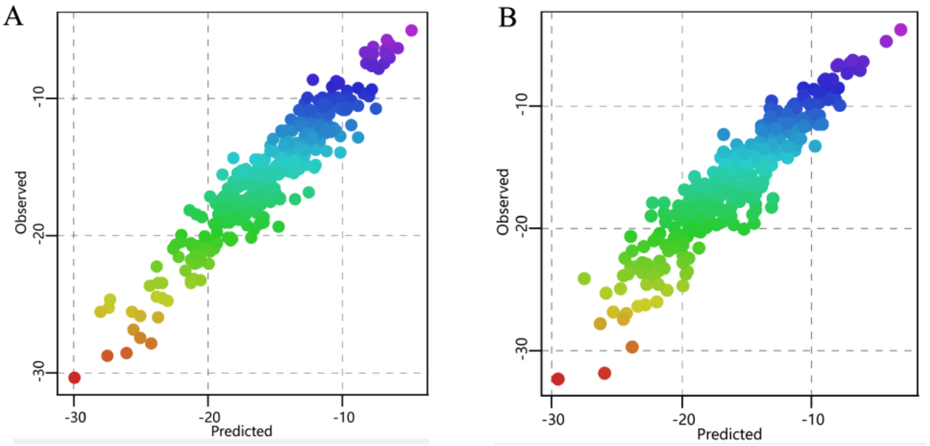

Figure S1. plot of predicted ΔG versus experimental ΔG of the best model in CDs delivery system for the training set (A) and the test set (B).

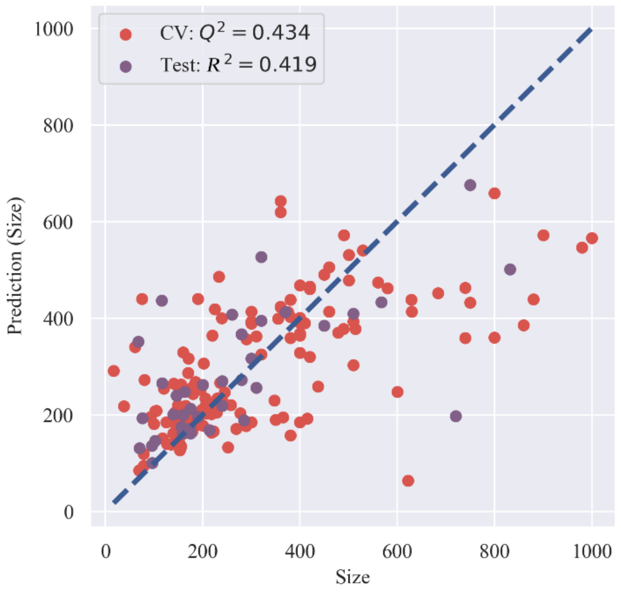

Figure S2. plot of predicted size versus experimental size of the best model in nanocrystal delivery system (using ASP preparing method) for the cross-validation and the test set.

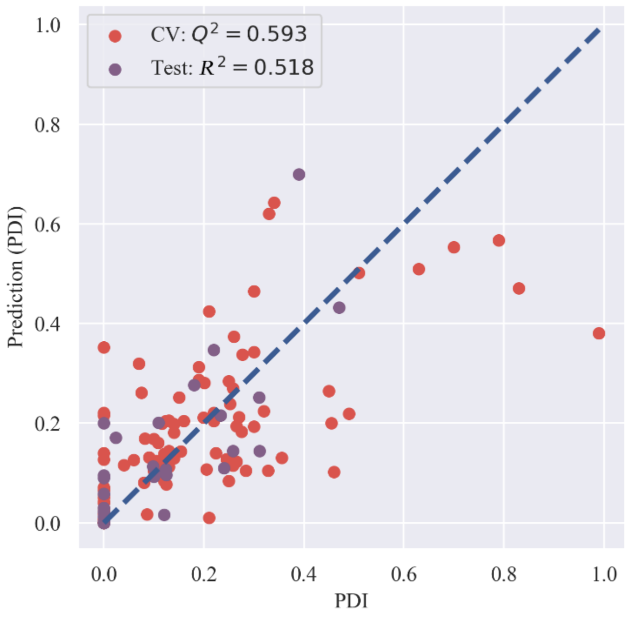

Figure S3. plot of predicted PDI versus experimental PDI of the best model in nanocrystal delivery system (using ASP preparing method) for the cross-validation and the test set.

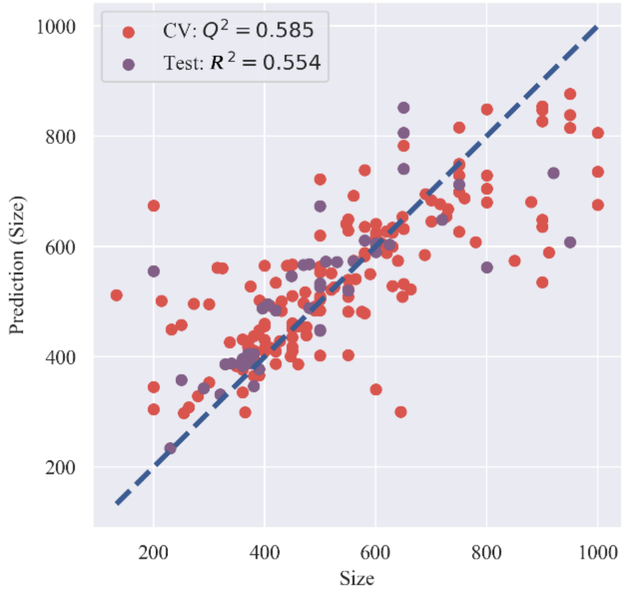

Figure S4. plot of predicted size versus experimental size of the best model in nanocrystal delivery system (using HPH preparing method) for the cross-validation and the test set.

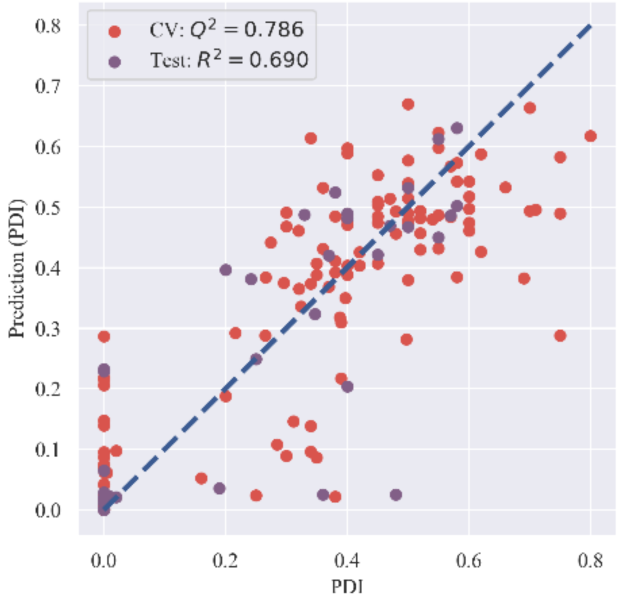

Figure S5. plot of predicted PDI versus experimental PDI of the best model in nanocrystal delivery system (using HPH preparing method) for the cross-validation and the test set.

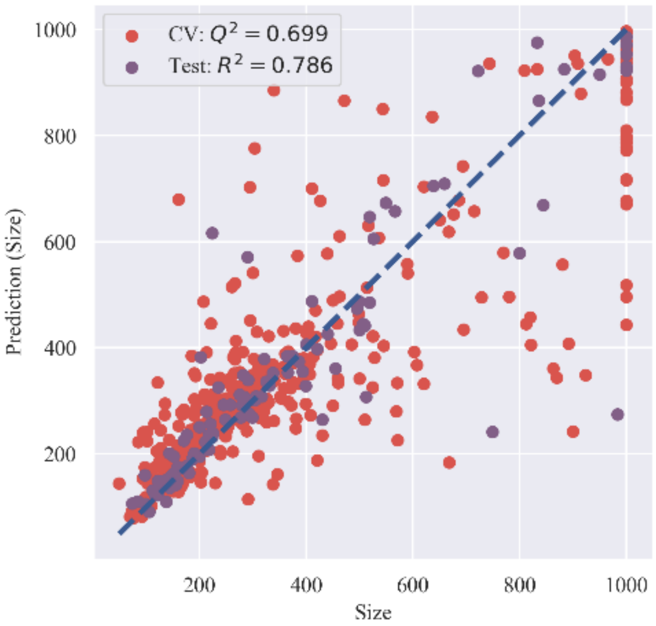

Figure S6. plot of predicted size versus experimental size of the best model in nanocrystal delivery system (using BWM preparing method) for the cross-validation and the test set.

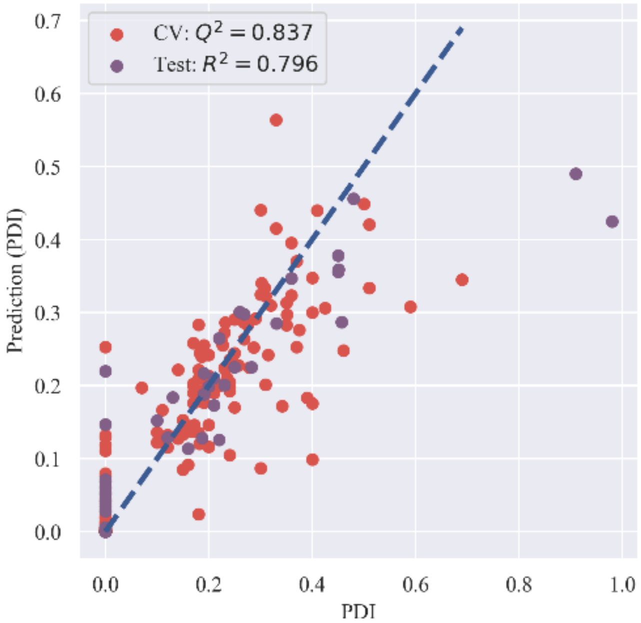

Figure S7. plot of predicted PDI versus experimental PDI of the best model in nanocrystal delivery system (using BWM preparing method) for the cross-validation and the test set.

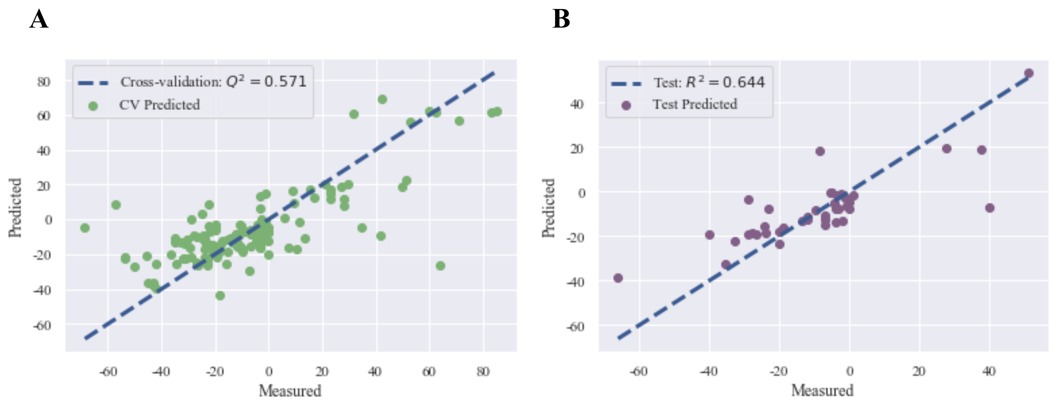

Figure S8. plot of predicted zeta values versus experimental values of the best model in liposome delivery system for the cross-validation (A) and the test set (B).

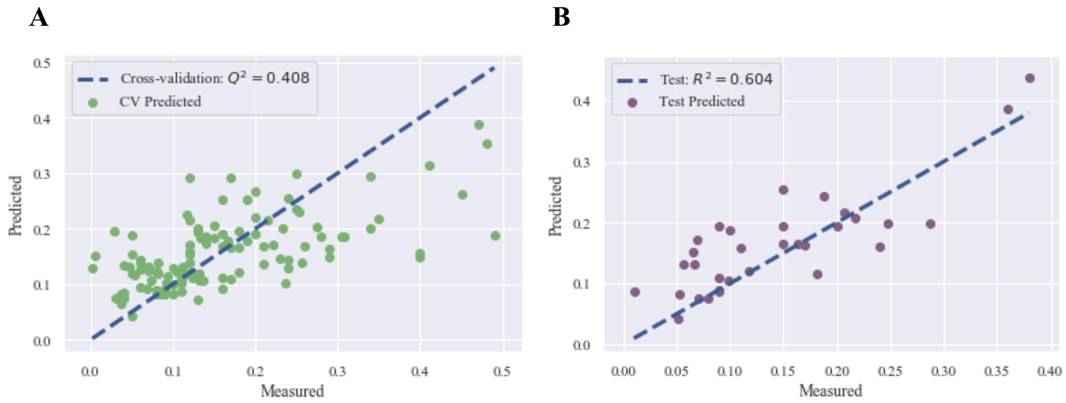

Figure S9. plot of predicted PDI values versus experimental values of the best model in liposome delivery system for the cross-validation (A) and the test set (B).

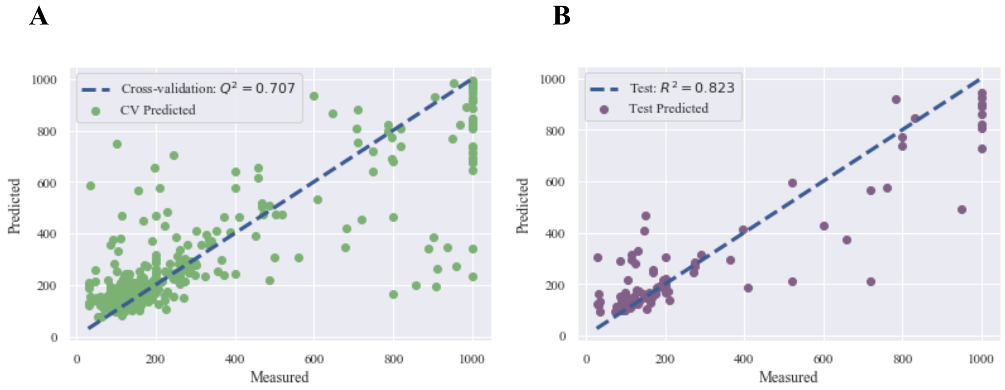

Figure S10. plot of predicted size values versus experimental values of the best model in liposome delivery system for the cross-validation (A) and the test set (B).

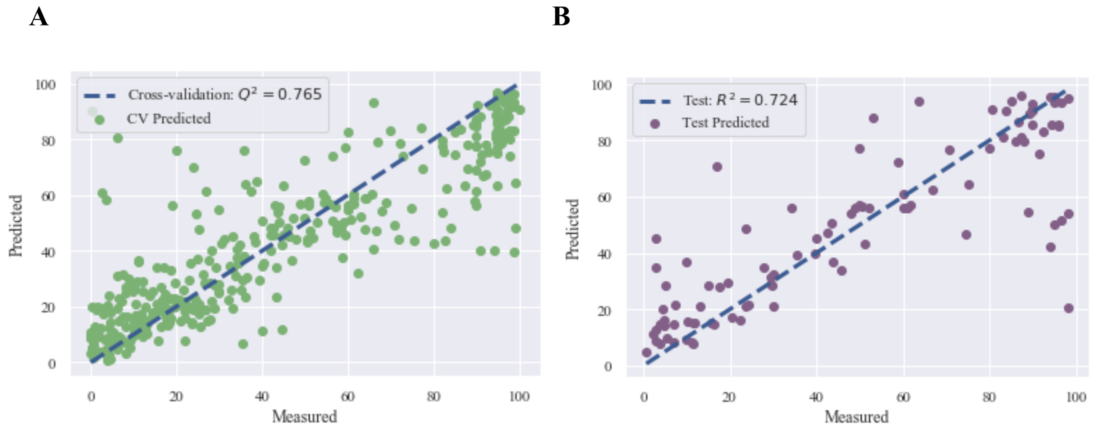

Figure S11. plot of predicted Encap (encapsulation) values versus experimental values of the best model in liposome delivery system for the cross-validation (A) and the test set (B).

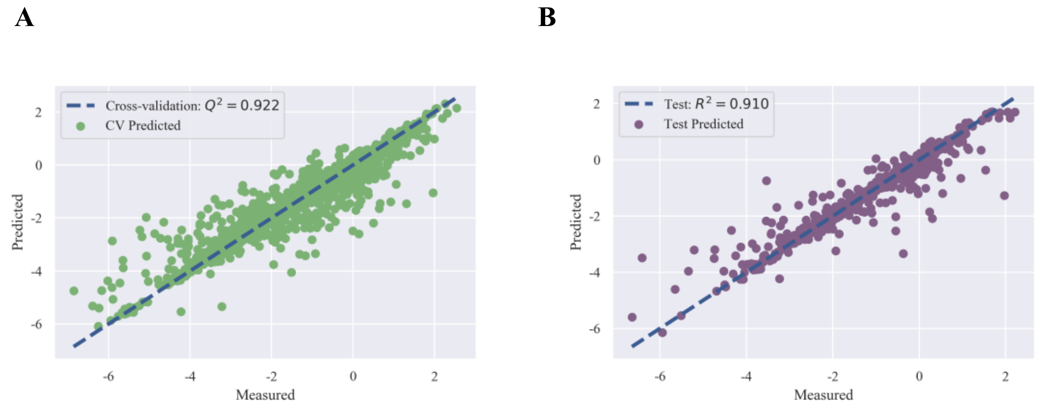

Figure S12. plot of predicted solubility values versus experimental values of the best model for the cross-validation (A) and the test set (B).

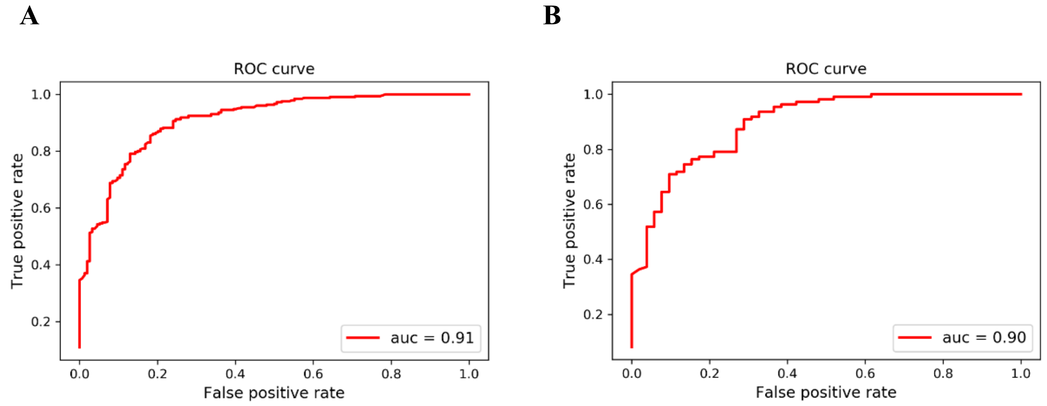

Figure S13. plot of ROC curves of the best model for 3-month stability evaluation in SD delivery system for the cross-validation (A) and the test set (B).

A B

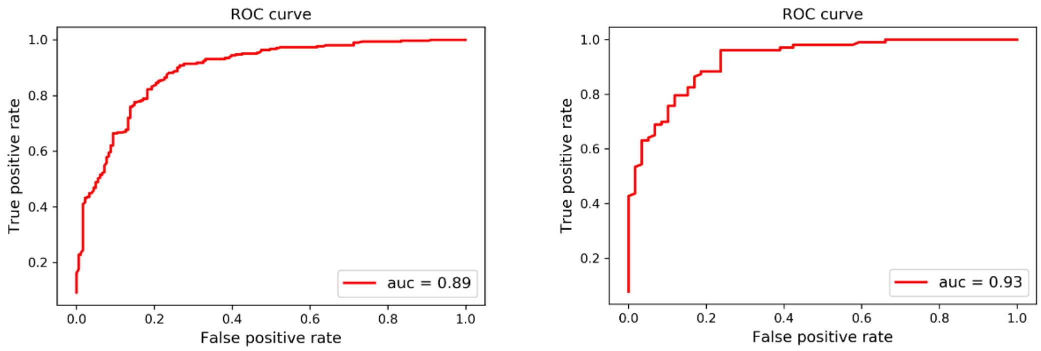

Figure S14. plot of ROC curves of the best model for 6-month stability evaluation in SD delivery system for the cross-validation (A) and the test set (B).

A B

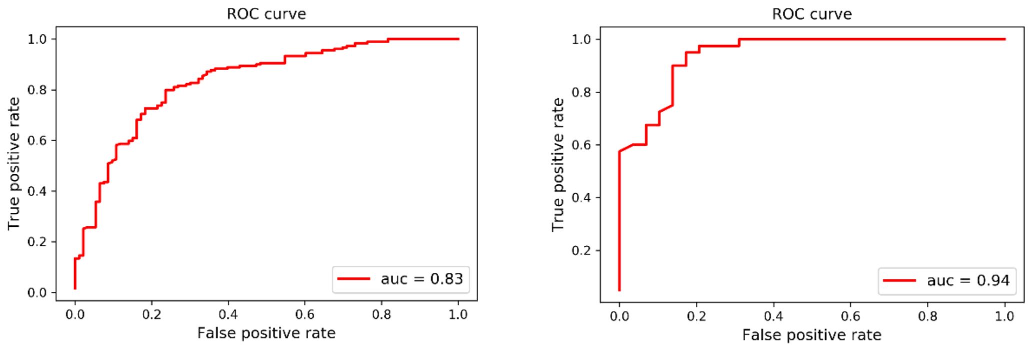

Figure S15. plot of ROC curves of the best model for prediction of complexation rate in phospholipid complex delivery system for the cross-validation (A) and the test set (B).

A B

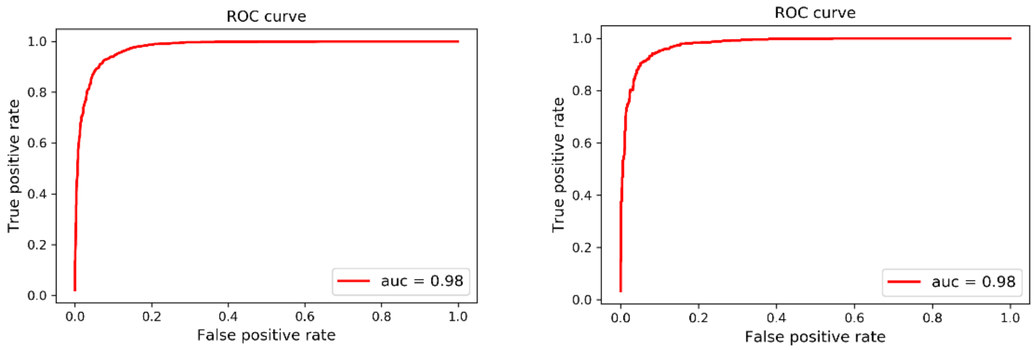

Figure S16. plot of ROC curves of the best model for prediction of self-emulsion status in self-emulsifying delivery system for the cross-validation (A) and the test set (B).

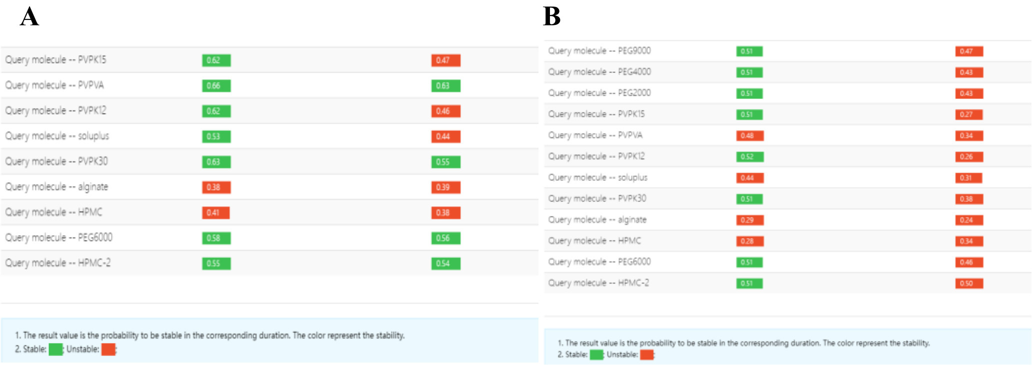

Figure S17. Prediction results of the 3-month and 6-month stability of formulation of ESTRADIOL and PVP K30 using a drug loading ratio of 1:2 (A) and 1:5 (B).

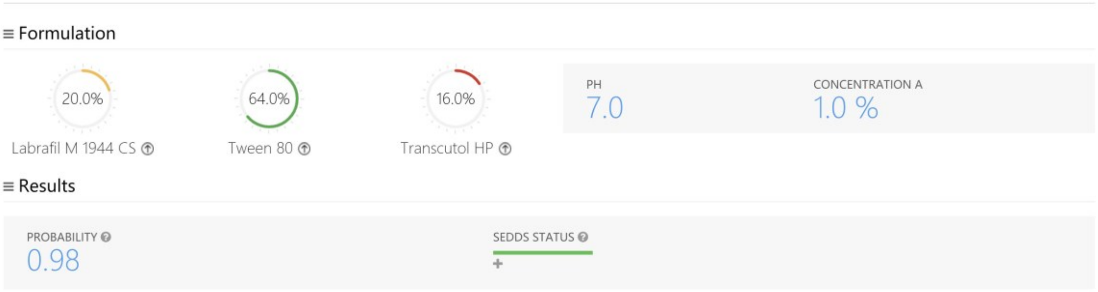

Figure S18. The predicted results of Labrafil M 1944 CS: Tween 80: Transcutol HP = 20: 64: 16 in pH 7.0 and concentration 1%.
